# Chronic use of diazepam in primary healthcare centers: user profile and usage pattern

**DOI:** 10.1590/S1516-31802007000500004

**Published:** 2007-09-02

**Authors:** Carmen Sylvia Ribeiro, Renata Cruz Soares Azevedo, Viviane Franco da Silva

**Keywords:** Diazepam, Benzodiazepines, Anti-anxiety agents, Primary health care, Drug prescriptions, Diazepam, Benzodiazepinas, Ansiolíticos, Atenção primária, Prescrição de medicamentos

## Abstract

**CONTEXT AND OBJECTIVE:**

Chronic use of benzodiazepines is frequent in general practice. The aim of this study was to describe the usage pattern and profile of chronic users of diazepam who had been consuming this drug for a minimum of thirty-six months continuously.

**DESIGN AND SETTING:**

This was a descriptive study (survey and clinical assessment) at five primary healthcare centers in Campinas, Brazil.

**METHODS:**

Psychotropic drug control books revealed 48 eligible patients. Among these, 41 were assessed by means of the Schedule for Clinical Assessment in Neuropsychiatry (SCAN), the Hospital Anxiety and Depression scale (HAD) and a questionnaire on usage pattern.

**RESULTS:**

Most patients were women (85.4%). The patients’ mean age was 57.6 years, and they were from the social strata C (39%), D (54%) and E (7%). The mean length of diazepam consumption was 10 years. The patients presented a lack of prescription compliance and had made frustrated attempts to stop using the drug. 55.5% said their doctor had never given any guidance on the effects of the drug. According to SCAN, 25 patients (61%) suffered from depressive disorders; only 12 cases of benzodiazepine dependence were detected by this instrument.

**CONCLUSION:**

There is a need to improve the detection and treatment of mental disorders, as well as to prevent inappropriate prescription and use of benzodiazepines. Diazepam dependence has distinctive characteristics that make it undetected by SCAN.

## RESUMO

**CONTEXTO E OBJETIVO:**

O uso crônico de benzodiazepínicos é freqüente. O objetivo deste estudo foi descrever o padrão de uso e o perfil dos usuários crônicos de diazepam (uso contínuo por no mínimo 36 meses) atendidos em unidades básicas de saúde.

**TIPO DE ESTUDO E LOCAL:**

Estudo descritivo, com inquérito e avaliação clínica padronizada, realizado em cinco unidades básicas de saúde de Campinas, São Paulo.

**MÉTODO:**

Os livros de controle de dispensa de psicofármacos revelaram 48 pacientes elegíveis, 41 dos quais foram avaliados por meio do Schedule for Clinical Assessment in Neuropsychiatry (SCAN), pela escala Hospital Anxiety and Depression (HAD) e de um questionário sobre padrão de uso.

**RESULTADOS:**

A maioria dos usuários era de mulheres (85,4%), com média de idade de 57,6 anos, pertencentes aos estratos socioeconômicos C (39%), D (54%) e E (7%). O tempo médio de uso de diazepam foi de 10 anos. Havia inobservância em relação à forma aconselhada de se utilizar o diazepam, bem como tentativas frustradas de parar a medicação. 55.5% afirmaram que não receberam orientações de seus médicos sobre os efeitos do medicamento. De acordo com o SCAN, 25 pacientes (61%) sofriam de depressão; apenas 12 casos de dependência a benzodiazepinas foram detectados por tal instrumento.

**CONCLUSÃO:**

São necessárias medidas que possam aprimorar a detecção e tratamento de transtornos mentais, assim como prevenir a prescrição e uso inadequado de benzodiazepinas. A dependência de bezodiazepínicos tem características que a difereciam de outras dependências químicas e não é detectada pelo SCAN.

## INTRODUCTION

Benzodiazepines are drugs with an anxiolytic effect, based on chlordiazepoxide synthesis, that started to be used in the 1960s. Their proven safety in comparison with barbiturates has contributed towards increased prescription rates since the 1970s.^1,2^ In the United States, 10-15% of adults take benzodiazepines at some time over a 12-month period and 2% use them chronically.^2^ Studies conducted in Chile, Italy and Spain have revealed prevalence of use of between 4 and 7.5% over the past 12 months.^3–5^ A figure of 10.2% has been found for the city of São Paulo, Brazil.^6^ A higher rate of 21.3% has been found in Porto Alegre, in southern Brazil.^7^ Besides high prevalence rates, studies have also indicated similarities in user profile, such that they are especially used among women and the elderly.^8–12^

One important matter to be considered regarding benzodiazepine consumption is physicians’ prescription habits, in addition to the usage pattern adopted by patients and their clinical profile. Some problems such as cognitive loss even after interruption, tolerance development, withdrawal symptoms and dependence have been associated with the chronic use of benzodiazepines.^13–17^ The problem becomes worse among the elderly because of age-related problems such as frequent comorbidities, use of several medications and pharmacodynamic as well as pharmacokinetic alterations.^18–20^

## OBJECTIVE

The aim of this study was to describe the usage pattern for diazepam provided by pharmacies at primary healthcare centers (PHCCs) and evaluate the profile of users who had been consuming this drug for a minimum of thirty-six months continuously.

## METHOD

### Type of study

This was a descriptive study in which chronic users of diazepam were surveyed and assessed by means of a standardized psychiatric instrument.

### Setting and subjects

This study was conducted in the northeastern region of the city of Campinas (State of São Paulo) in 2001. This region had 152,438 residents at that time (15.7% of the population of the city), and was characterized by its poor economic status and high unemployment rates. It had eight PHCCs, four of which had a mental health team. Five PHCCs were included in this study because they were the first to implement the use of psychotropic drug control books. These books revealed that 1,458 individuals had received diazepam at some time over the past three years.

The inclusion criteria for the present study were that the patients should be over 15 years old and among these 1,458 individuals, who had been using diazepam for a minimum of thirty-six months continuously, according to the monthly records in the books. These monthly records were, in turn, in accordance with the prescriptions made by the patients’ doctors. Furthermore, the patients had to confirm that they were the users of the benzodiazepines provided to them by the PHCC pharmacy. Accordingly, 48 individuals were eligible (the other 1410 had not taken diazepam for a minimum of thirty-six months continuously). It was possible to evaluate 41 of them (six patients did not respond to the three recall invitations made and one patient had moved to another town).

### Instruments and variables

The following data collected from the psychotropic drug control books were stored in an electronic file: total annual consumption of diazepam; average monthly consumption; mean duration of use; average daily dosage; and prescribing physician's specialty.

The clinical assessment of the patients involved:

1. A questionnaire to assess socioeconomic status based on ownership of items and the reported educational level of the head of the family. This instrument included five social strata, A to E, defined by score ranges.^21^ These strata were in accordance with the definitions of the Instituto Brasileiro de Geografia e Estatística (IBGE).
2. The Hospital Anxiety and Depression scale (HAD). This consisted of 14 multiple choice questions within two subscales: depression and anxiety. The overall score for each subscale ranged from 0 to 21.^22^ Patients whose score was more than eight in the respective HAD subscales were considered to be possible cases of anxiety and depression, in accordance with the national validation study.^23^
3. The Schedule for Clinical Assessment in Neuropsychiatry (SCAN). This consisted of a sequence of tools that identified, measured and classified psychopathological conditions and also behavior associated with psychiatric disorders in adults.^24^ Besides the formal psychiatric diagnosis, the second section of SCAN consisted of a list of physical symptoms that were not related to any specific physical condition but were to be ticked according to the interviewer's judgment. The SCAN system had two essential elements: a glossary of definitions and CATEGO (a data processing computer program).
4. A questionnaire on the diazepam usage pattern was drawn up for this study, composed of open questions and questions with multiple-choice answers in two domains: the patient's habits and difficulties regarding chronic use of the medication and the patient's opinion about the prescription and the physician's prescribing behavior.

### Procedure

The patients received letters explaining the objectives and procedures of the study and recalling them to the PHCC. After they signed an informed consent form, the interview was carried out. A report on the patient's condition revealing diazepam dependence was discussed with the attending physician and attached to the respective file. The first author of the present study (C.S.R), who underwent training on applying SCAN at a World Health Organization accredited center, conducted all the interviews. This research protocol was approved by the Ethics Committee of the Medical School of Universidade Estadual de Campinas.

### Data analysis

The data were organized in frequency tables with the respective percentages. Means and standard deviations were calculated for continuous variables. SCAN data were processed using the CATEGO system.^24^

## RESULTS

Most of the patients who had been using diazepam continuously for 36 months were women (85.4%) and white (85.4%). The patients’ mean age was 57.6 years (standard deviation, SD = 12.5) and only four patients were below the age of 40 years; 43.9% were married and 70.8% were Catholics. The patients were from the social strata C (39%), D (54%) and E (7%).

The mean duration of diazepam consumption was 10 years (SD = 7.95). The patients began using diazepam at an average age of 43 years (SD = 14) and generally (73.2 %) took a single nocturnal dose of 10 mg. Thirty-seven (90.2%) out of the 41 patients used some other medication besides diazepam: mainly antihypertensives, antiarrhythmics and antidepressives (26, 8 and 7 patients, respectively). Ten patients (24.4%) used three or more drugs besides diazepam.

Table 1 demonstrates the percentages of affirmative responses to the items in the questionnaire on usage patterns. When an attempt to stop using the drug was made, at least one withdrawal symptom was highlighted. Among the withdrawal symptoms remembered by the patients when answering the item “Report what you felt ……”, the most common ones were insomnia (46.3%), agitation (9.7%), headaches or pain in the limbs (9.7%) and irritability (9.7%). Six of the seven patients who reported taking diazepam overdoses had needed urgent medical care. Ten of the 30 patients who tried to stop taking diazepam were motivated by their physicians to do so.

**Table 1.** Pattern of benzodiazepine usage among patients in public primary healthcare centers

	n = 41	%
Significant withdrawal symptoms	36	87.8
Frustrated attempts to stop consumption	30	76.9
If the drug was not supplied, patient would pay for it	17	43.6
Habit of lending the medication to others	17	41.5
Lack of compliance regarding prescription*	16	39.0
Preoccupations about chronic effects of diazepam	13	30.2
“Suggestion” of diazepam use to others	06	15.0
Intake of several pills at the same time	06	14.6

* *Negative answer to the question “Have you used diazepam according to your physician's prescription?”*

The reasons reported for beginning and continuing to use diazepam can be seen in Table 2 (spontaneous answers to the open questions: “Why do you think your physician prescribed diazepam?” and “Why did you continue using diazepam?”).

**Table 2.** Reasons for starting to use diazepam and continuing with it, as reported by the patients of public primary healthcare centers

	n = 41	%
**Main reason for starting**		
Nervousness	17	41.4
Insomnia	15	36.6
Fear/panic	4	9.8
Fainting	3	7.3
Hypertension	2	4.9
**Main reason for continuing**		
Insomnia	10	24.4
Calming effect	9	21.9
Dependence	5	12.2
Depression	3	7.3
Fainting	1	2.4
Heart problems	1	2.4
Cramps in the limbs	1	2.4
Hypertension	1	2.4
”I don't know why”	10	24.4

The specialty of the prescribing physician was: general clinician (44.4% of the cases), psychiatrist (41.7%), cardiologist (5.6%), neurologist (5.6%) and gynecologist (2.8%). With regard to the guidance given by the prescribing physician for the patient's health problem, 22.5% of the patients reported they had received guidelines but did not understand them, 55.5% affirmed that they had never received any guidance and only 15% (eight patients) remembered some information received from the doctor. According to the HAD scale, 64.1% of the cases presented anxiety and 69.2% presented depression.

Table 3 shows the ten most common physical and emotional symptoms verified by SCAN. Table 4 presents a list of the psychiatric diagnoses based on the International Classification of Diseases, 10^th^ edition (ICD-10), that were obtained from SCAN. This only detected 12 cases of benzodiazepine dependence.

**Table 3.** Schedule for Clinical Assessment in Neuropsychiatry (SCAN) frequencies for physical and emotional symptoms among diazepam users

Description of symptoms	%
**Physical symptoms**	
Pain in the arms, legs, joints, hands and feet	78.4
Problems with sight, hearing and speech	64.9
Palpitations, heart beating without stopping	64.9
Dry mouth	63.2
Memory loss	58.3
Dormancy, cramps	56.8
Short respiration with no physical exercise	54.1
Hot and cold flushes	48.6
Headaches	43.2
Gastrointestinal complaints	40.5
**Emotional symptoms**	
Depressive mood	52.6
No hope for the future	52.6
Preoccupation	48.6
Generalized/localized muscular tension	43.2
Lack of energy	39.5
Initial insomnia	39.5
Satisfactory quality of sleep	39.5
Feelings of psychomotor retardation	36.8
Unresponsive depression	34.2
Accentuated lack of pleasure	31.6

**Table 4.** Frequency of mental disorders according to the International Classification of Diseases, 10^th^ edition (ICD-10), among diazepam users

Diagnostic category*	Male (n = 6)	Female (n = 35)	% (n = 41)
Recurrent depressive disorder (F33)	1	12	31.7
Mental disorders caused by the use of sedatives and hypnotics (F13)	1	11	29.2
Dysthymia (F34.1)	0	10	24.3
Sleep disorder (G47)	0	6	14.6
Generalized anxiety disorder (F41.1)	0	3	7.3
Somatoform disorder (F45)	1	2	7.3
Mental disorder caused by a brain lesion (F06)	0	2	4.8
Depressive episode (F32)	0	2	4.8
Schizoaffective disorder, depressive type (F25.1)	1	0	2.4
Bipolar affective disorder in remission (F31.7)	1	0	2.4
Obsessive-compulsive disorder (F42)	0	1	2.4
Intentional exposure to analgesics, antipyretics and antirheumatics (X60)	0	1	2.4

* *More than one diagnostic category was given to some patients.*

## DISCUSSION

Studies that specifically report on benzodiazepine consumption at PHCCs are rare.^25–27^ Our study provided an estimate of the diazepam usage pattern as well as the patients’ clinical profile.

Some methodological limitations should be taken into consideration. The first of these was the lack of a precise instrument for evaluating benzodiazepine dependence, since the SCAN criterion for benzodiazepine dependence has proved to have little sensitivity. Other studies^28,29^ have already suggested that the operational criteria used in the diagnostic assessment of psychoactive substance dependence have proved to be useless with regard to benzodiazepines. The benzodiazepine dependence indicators suggested by a Canadian study were: self-identification as a dependent, enumeration of multiple stressors to justify use, use based on anticipated stress, minimization of damage, storage of medication and failure to stop or diminish use.^29^ Our findings reinforce the need for greater precision in establishing operational criteria for benzodiazepine dependence.

With regard to the patients’ sociodemographic profile, it is important to remember that our study comprised patients seen at PHCCs, within the public heath system. There were no individuals belonging to socioeconomic levels A and B. Both Brazilian and international studies agree that women aged over 40 years and elderly men present the highest prevalence of benzodiazepine use.^2,6,8,17,25–30^ However, it should be borne in mind that our sample came from a region of the city of Campinas in which the residents are predominantly in lower middle and poor social classes, and are, therefore, public service users, and this impedes data generalization.

The overall trend was for individuals to use constant doses of benzodiazepine, with no progressive increases in dosage. Our findings regarding the reasons why the first prescription was issued (agitation and insomnia) were in accordance with other studies.^30–32^

The patients’ clear lack of understanding regarding the physician's instructions may be partly explained by their poor level of schooling. In addition to the specific maintenance prescription treatment, the physician ends up simply repeating previous prescriptions, thereby perpetuating a practice that is not always based on formal therapeutic criteria. Repeated prescriptions at each visit, together with the patient's personality characteristics, may increase the risk of benzodiazepine dependence.^30–34^

In the present study, 25 patients (61%) suffered from depression, according to the diagnoses produced by SCAN. The frequency with which depressive symptoms appear in the list of most reported emotional symptoms was also noticeable (it is worth clarifying that the patients were asked about each symptom in the SCAN list). On the other hand, there was no spontaneous report of depression as the main reason for having started using benzodiazepine. It is known that many patients suffering from depression who are seen by general practitioners are given only benzodiazepine for anxiety symptoms and insomnia, instead of the appropriate treatment for depression. Quality of care in cases of depression depends on good communication between the doctor and the patient, but patients who are depressed often have difficulty in discussing their problems with doctors. They are also unlikely to be active in seeking care and have low expectations of what the treatment can provide.^31,32^

According to our findings, the chronic benzodiazepine users at PHCCs did not have the required information and consequently did not have any preoccupations about the appropriateness of their treatment. When they did, they were unable to stop taking benzodiazepines without presenting withdrawal-related symptoms.

There is a need for guidance and follow-up for chronic users of benzodiazepine, especially in view of the low cost and effectiveness of the intervention programs for benzodiazepine-dependent patients. One very simple and straightforward program showed the positive economic implications of sending an educational letter to patients, aimed at reducing long-term benzodiazepine prescribing. After receiving the letter, 31% of those patients discussed their benzodiazepine usage with their general practitioner and 10% had their drug or drug strength changed. During the year following this intervention, a significant reduction in benzodiazepine usage of 17% was observed in relation to baseline; 5% of the patients did not order any more benzodiazepine prescriptions after receiving the letter.^31^ There is also a need to improve medical students’ and professionals’ skills regarding the detection and management of anxiety and depressive disorders.

## CONCLUSION

Chronic benzodiazepine users are frequently women over the age of 40 years, who began using the medication because of nervousness or insomnia. The usage pattern highlighted the patients’ lack of prescription compliance, frustrated attempts to stop using this drug and frequent chronic concomitant use of one or more other drugs. SCAN proved to be inadequate for detecting benzodiazepine dependence syndrome. Most patients (61%) presented with depression.
